# 20 Hz Transcranial Alternating Current Stimulation Inhibits Observation-Execution-Related Motor Cortex Excitability

**DOI:** 10.3390/jpm11100979

**Published:** 2021-09-29

**Authors:** Lijuan Wang, Michael A. Nitsche, Volker R. Zschorlich, Hui Liu, Zhaowei Kong, Fengxue Qi

**Affiliations:** 1 School of Sports Medicine and Rehabilitation, Beijing Sport University, Beijing 100084, China; wanglijuan@bsu.edu.cn; 2 Department of Psychology and Neurosciences, Leibniz Research Centre for Working Environment and Human Factors, 44139 Dortmund, Germany; nitsche@ifado.de; 3 Department of Neurology, University Medical Hospital Bergmannsheil, 44789 Bochum, Germany; 4 Institute of Sport Science, Carl von Ossietzky Universität Oldenburg, 26129 Oldenburg, Germany; volker.zschorlich@uni-rostock.de; 5 Institute of Sport Science, University of Rostock, 18057 Rostock, Germany; 6 China Institute of Sport and Health Science, Beijing Sport University, Beijing 100084, China; liuhuibupe@163.com; 7 Faculty of Education, University of Macau, Taipa, Macao 999078, China; zwkong@um.edu.mo; 8 Sports, Exercise and Brain Sciences Laboratory, Beijing Sport University, Beijing 100084, China

**Keywords:** transcranial alternating current stimulation, motor cortex excitability, action observation, action execution

## Abstract

The present study aimed to investigate the effect of transcranial alternating current stimulation (tACS) on the primary motor cortex (M1) during action observation, and subsequent action execution, on motor cortex excitability. The participants received tACS at 10 Hz or 20 Hz, or a sham stimulation over the left M1 for 10 min while they observed a video displaying a repeated button-tapping task using the right hand, and then performed an identical task with their right hand. Motor-evoked potential (MEP) amplitudes were measured before (T0) and after the action observation paired with tACS or a sham stimulation (T1), and after the performance of the action (T2). The results showed that MEPs were significantly reduced at time point T1 (*p* = 0.042, Cohen’s *d* = 0.611) and T2 (*p* = 0.0003, Cohen’s *d* = 0.852) in the 20 Hz tACS condition, in contrast with the sham stimulation. There was a significantly smaller MEP amplitude at time point T2 in the 20 Hz tACS condition, as compared to the 10 Hz tACS condition (*p* = 0.01, Cohen’s *d* = 0.622), but the MEP amplitude did not significantly change at time point T1 between the 20 Hz and 10 Hz tACS conditions (*p* = 0.136, Cohen’s *d* = 0.536). There were no significant differences at time point T1 and T2 between the 10 Hz tACS condition and the sham stimulation. We conclude that 20 Hz tACS during action observation inhibited motor cortex excitability and subsequently inhibited execution-related motor cortex excitability. The effects of tACS on task-related motor cortex excitability are frequency-dependent.

## 1. Introduction

Functional neuroimaging studies provide evidence that the action-observation and the execution-related cortical network includes the primary motor cortex (M1), the ventral premotor cortex, the primary somatosensory cortex, and the inferior frontal gyrus [1,2]. Cortical activity in these networks is enhanced when individuals perform a movement or observe an identical movement performed by another individual [3]. The alpha (8–13 Hz) and beta (14–30 Hz) components of the mu rhythm desynchronize within these networks when preparing, executing and controlling voluntary movements, as well as action observation [4,5]. Neuroscientific research further shows that alpha oscillations are associated with perception, memory, attention, and execution. The increased power of alpha oscillations reflects the inhibition of task-irrelevant brain areas, while the attenuation of alpha oscillations indicates task-relevant neuronal processing [6,7,8,9]. Beta band oscillations are considered “antikinematic”, since beta oscillations are prominent during tonic motor output but desynchronize during voluntary movement preparation, execution, and motor learning [7,10].

The brain is a complex adaptive system that could be flexibly adaptive to the changing environment. Structural, functional and effective connectivity are three interrelated forms of connectivity that are involved in the brain network, forming the basic concept of neuroplasticity [11]. Non-invasive brain stimulation techniques may induce neuroplastic changes in brain areas [11]. Transcranial electrical stimulation is suitable for the modulation the activity of the respective brain networks and the alteration of task-related physiological processes, as well as task performance. Transcranial alternating current stimulation (tACS) involves the application of weak non-invasive oscillating currents over the cortex to alter neural excitability [12]. Frequency-specific entrainment of endogenous brain oscillations and the modulation of spike-timing-dependent plasticity (STDP) has been proposed to be the physiological and working mechanism of tACS [11,13]. A study by Antal and Paulus (2012) showed that neuroplastic changes are the mechanism of tACS after-effects [14]. Furthermore, tACS modulates functional connectivity that, theoretically, might be applied to change emergent properties of network function [11]. Several studies in non-human primates have demonstrated that tACS entrains activity on a single-neuron level in a frequency-specific way and affects neural spike timing in a dose-dependent manner [15,16]. Weak sinusoidal voltages were shown to elicit spiking activity, and alternating current stimulation at the frequency of endogenous oscillations mainly affects spike timing. The effect of tACS may be explained via the synaptic plasticity modulated by STDP [13]. Human studies with simultaneous electroencephalographic and magnetoencephalographic recordings also show entrainment of brain oscillations by tACS [17,18]. The behavioral effects of tACS have thus been related to the neural entrainment of ongoing oscillatory brain activity to the respective stimulation frequency [9]. Transcranial alternating current stimulation modulates intrinsic brain oscillations via interference with endogenous cortical rhythms when the stimulation frequency matches the natural rhythm of the stimulated brain region [9]. Transcranial alternating current stimulation applied at 15 Hz and 20 Hz with an intensity of 1 mA, delivered over M1 for 10 or 20 min decreased motor cortex excitability [13,19,20] and slowed down voluntary movement [13] in some studies. One possible mechanism of action is that beta oscillations are linked to the balance between GABAergic and glutamatergic input [21]. The enhancement of GABAergic activity is associated with higher resting beta power and beta desynchronization during movement-related processes [12,22]. Other studies, however, suggest that 20 Hz tACS over M1 for 15 min with 1.5–2 mA of stimulation intensity increases motor cortex excitability [23,24]. Wischnewski et al. (2019) reported that a 2 mA stimulation protocol increased motor-evoked potential (MEP) amplitudes under placebo stimulation, but this effect was blocked under the N-methyl-D-aspartate receptor (NMDAR) antagonist dextromethorphan. This dependency of the excitability-enhancing effect on glutamatergic receptor activity is in line with the mechanism of STDP [23]. The discrepancies between study results may be attributed to the different current intensities of tACS applied in the respective experiments. In the studies of Wischnewski et al. (2019) and Gallasch et al. (2018), the current intensities were 2 mA and 1.5 mA, respectively [23,24], while in studies that reported an inhibitory effect in motor cortex excitability, 1 mA was used as the intervention intensity [19,20]. This mechanistic explanation is in line with observations showing that high current intensity induces excitatory effects, while low current density induces inhibitory effects [25]. The diverse activation thresholds of different pyramidal cell sub-populations might contribute to the inverse effect of different current intensities [25]. By contrast, 10 Hz tACS continuously delivered over M1 for a duration of 2 to 10 min did not induce changes in motor cortex excitability [19,26]. This might be explained by the fact that alpha oscillations mainly be located in the primary somatosensory cortex and are more associated with sensory processes [27].

In this study, we contrasted the effect of tACS at an intensity of 1 mA and sham stimulation over M1 on motor cortex activity during action observation and subsequent action execution. Since the desynchronization of beta oscillations in motor cortical regions are associated with voluntary movement, we expected that 20 Hz tACS over M1 for 10 min during action observation would synchronize local cortical oscillatory activity and/or induce excitability-diminishing plasticity, thus reducing motor cortical excitability, and that this effect would remain during subsequent action execution. Alpha oscillations predominantly reflect visuospatial-related parameters during action observation [28] and are located in the primary somatosensory cortex during motor tasks [29]; therefore, we hypothesized that tACS at 10 Hz would not influence motor cortex excitability during the observation or subsequent performance of actions.

## 2. Materials and Methods

### 2.1. Participants

Twenty-eight healthy adults (mean age, 24.29 ± 3.30 years; 10 females) gave written informed consent to participate in this study. The participants were randomly assigned to tACS at 10 Hz and 20 Hz and/or a sham stimulation and participated in the experimental conditions described below. Fifteen participants performed only one condition, six subjects participated in two conditions, and seven participants performed in three conditions. Each stimulation condition was performed by 16 participants. To prevent carryover effects, all the experimental conditions were separated by at least 7 days (10.40 ± 6.87 days). Three participants were separated by 21, 27, and 28 days between two experimental conditions, respectively, because of personal reasons. None of the participants were pregnant, had metal implants, a history of orthopedic disorders, central nervous system disease, neurological or psychiatric disease, or other medical diseases. All participants had normal or corrected-to-normal vision. According to the Oldfield’s Edinburgh Handedness Inventory [30], only right-handed participants were recruited. The study was approved by the ethics committee of the University of Rostock and conformed to the standards of the Declaration of Helsinki.

### 2.2. Transcranial Magnetic Stimulation

Single-pulse bi-phasic transcranial magnetic stimulation (TMS) over the left M1 was performed using a D-B80 coil connected to a MagPro R100 magnetic stimulator (Medtronic, Skovlunde, Denmark) to monitor motor cortex excitability. The TMS coil was held tangentially to the scalp with the handle pointing backward and laterally angled at about 45° away from the midline. The heads of the participants were stabilized by a chin-forehead support to minimize head movement. The “hot spot” was defined as the optimal cortical representation of the right first dorsal interosseous (FDI) muscle, where the coil was moved in 0.5 cm steps at a moderately suprathreshold stimulation intensity to identify the coil position that consistently elicited the largest MEPs. The site was marked with a soft pen, and the handle was fixed by a mechanical arm (Manfrotto, Feltre, Italy) to ensure the correct position of the coil throughout the experiment. The TMS pulse was delivered at an intensity that evoked MEP amplitudes of approximately 1 mV (SI1mV) peak-to-peak at baseline. Twenty MEPs per time point were obtained with the respective TMS intensity throughout the experiment, and the interval between the TMS pulses was 4 s, with a jitter of ±0.5 s.

Surface electromyography (EMG) was recorded from the right FDI muscle via Ag-AgCl electrodes in a belly-tendon montage (Hellige Baby-Electrodes; GE Medical Systems, Milwaukee, WI, USA). A ground electrode was positioned over the right lateral biceps brachii muscle. The signals were amplified with an amplification rate of 1000 (Biovision, Wehrheim, Germany), and filtered with a 5 Hz digital second-order Butterworth high-pass filter. All the EMG signals were collected by DAQ-Card 6024, and were processed by DIAdem software (National Instruments, Austin, TX, USA).

### 2.3. tACS

In this single-blind study, tACS was delivered for a duration of 10 min with 5 s of ramp-up and ramp-down by a battery-driven electrical stimulator (BrainSTIM, EMS, Bologna, Italy) through a pair of surface saline-soaked sponge electrodes (5 × 5 cm^2^). The target electrode was centered over the left M1 representational site of the right FDI muscle, which was identified by TMS. The return electrode was positioned over the contralateral supraorbital region. Transcranial alternating current stimulation was applied at 10 Hz and 20 Hz, respectively, with a current intensity of 1 mA (peak-to-peak), with no direct current offset. For the sham stimulation, the current was turned on for 30 s, with 5 s of fade-in and fade-out, and then turned off. 

### 2.4. Action Observation and Execution

Participants kept their hands in a relaxed position and were seated comfortably in front of a computer screen (24 inches), located at 80 cm eye distance. Participants were instructed to watch a video displaying a right hand pressing buttons that were mapped horizontally in a box. The video lasted for 10 min and was composed of 20 short clips. Twenty-second-long clips were displayed at natural speed, and 40 s long clips displayed at half of natural speed were presented 10 times. A 20 s long clip was always followed by a 40 s long clip. In the video, four black spots were shown on the screen (from left to right 1, 2, 3, and 4, respectively). While the black spot turned into a red spot every 3 s in an orderly sequence (1, 3, 3, 1, and 4, respectively), a human right hand reached the box and pressed the appropriate button with the index finger (the other fingers shrank). The hand returned to the resting position immediately after pressing the button. Since attention affects motor cortical plasticity [31], the participants were asked to concentrate on the button-pressing task displayed on the screen and count the number of buttons pressed in the 10 min long video. At the end of the video, the participants verbally reported the counted amount of button presses.

After the action observation, the participants were asked to perform the same task that was displayed in the video. The participants were seated in front of a table with a 24-inch computer screen and a custom-made box with four red buttons (from left to right 1, 2, 3, and 4, respectively). Each of the red buttons corresponded to the black spots on the screen. In the beginning, the participants put their right hand at a 20 cm distance from the button box. The left hand was kept in a relaxed position throughout the experiment. The participants were asked to pay attention to the screen. When the black spots turned into red spots in a modeled response sequence (1, 3, 3, 1, and 4, respectively) every 3 s, the participants had to reach for the box and press the corresponding button with the index finger of their right hand immediately, and then move the right hand quickly back to its original position. The action execution task lasted for 160 s and the response sequence was repeated eight times.

### 2.5. Experimental Design and Procedures

**Figure 1:**
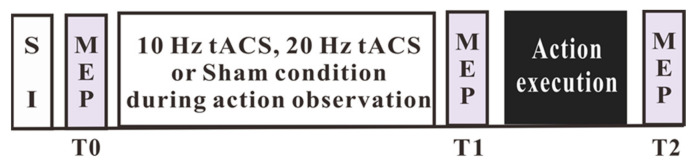
Timeline of the experimental design. Either tACS at 10 Hz and 20 Hz or a sham stimulation was applied over the left M1 during the action observation, followed by the action execution. Twenty motor-evoked potentials (MEPs) per time point (before, and after the action observation, and after the action execution) were obtained via a single-pulse TMS with an intensity that elicited MEP amplitudes of about 1 mV (SI1mV) at baseline.

Before the experiment, neurological examinations were performed to exclude patients with neurological diseases, and the Edinburgh Handedness Inventory was used to include only right-handed participants. The participants were informed about the experimental procedures upon arrival at the laboratory and signed an informed consent form. The participants were seated in a chair with their heads stabilized by a chin-forehead support. The TMS coil was positioned over the left M1, and the optimal position of the magnetic coil for activating the right resting FDI muscle was determined, as described above. Twenty MEPs were obtained with the respective TMS intensity for the determination of baseline motor cortex excitability (T0). The participants were then seated comfortably in front of a computer screen and watched the video displaying the button-pressing task for 10 min. During the action observation, the participants received 10 Hz tACS, 20 Hz tACS or sham a stimulation over the aforementioned “hot spot”. After the end of the action observation combined with the tACS or sham stimulation, 20 MEPs were again obtained via TMS with baseline intensity (T1). Afterwards, the participants performed the action execution task, and motor cortex excitability was again monitored via 20 MEPs elicited by TMS (T2). For an overview of the time course of the experiment, see Figure 1.

### 2.6. Statistical Analysis

SPSS (version 22.0; IBM, Armonk, NY, USA) and Prism (Version 8; GraphPad Software, San Diego, CA, USA) were used to perform the statistical analyses. In case of spontaneous muscle activity in the time window of 300 ms prior to TMS with an amplitude above 50 µV, the respective MEPs were discarded. The average peak-to-peak amplitudes of the 20 MEPs for each block were calculated individually and normalized to baseline. The differences in baseline MEP amplitudes between the experimental conditions were analyzed by one-way analysis of variance (ANOVA). The preliminary analyses of SI1mV showed heterogeneity of variance (one-way ANOVA) that could not be improved using standard transformations (base 10, natural logarithm). Therefore, the differences in SI1mV between the experimental conditions were analyzed by using the Kruskal-Wallis test.

The intervention-related alterations of MEP amplitudes were analyzed by using multi-level modeling analysis. The MEP amplitudes normalized by baseline served as the dependent variable. The fixed effects in this model included time (three time points: T0, T1, and T2), group (three stimulation conditions: 10 Hz tACS, 20 Hz tACS and sham stimulation), and the time by group interaction. The subject–level intercept was included in the model as a random effect. The Restricted Maximum Likelihood method was used to minimize underestimated variance. Different candidate models were compared and the fit of each model was evaluated by using Restricted Log Likelihood, Akaike’s Information Criterion (AIC), and Schwarz’s Bayesian Criterion (BIC). Fisher’s LSD post-hoc tests were performed to determine changes between the conditions. Cohen’s *d* was applied to calculate the effect sizes. The Shapiro–Wilk test was performed to explore the normal distribution of the data. The significance threshold was set to *p* < 0.05 for all the statistical tests.

## 3. Results

All the participants tolerated tACS well. The stimulation conditions did not differ significantly with respect to baseline SI1mV and MEP amplitudes (all values of *p* ≥ 0.422).

**Figure 2:**
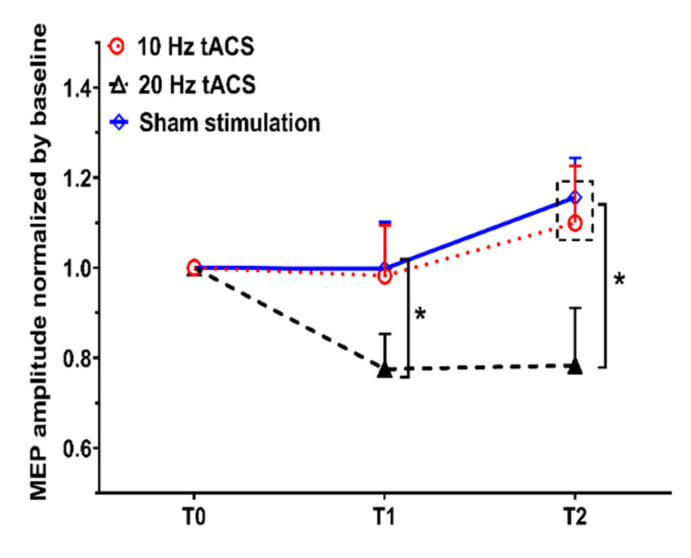
Impact of tACS on motor cortex excitability. Either tACS at 10 Hz and 20 Hz or a sham stimulation was applied over the left M1 during the action observation, followed by the action execution. Motor-evoked potentials (MEPs) were recorded before the action observation (T0), immediately after the action observation (T1), and after the action execution (T2). Each intervention condition included 16 participants. Error bars represent the standard error of means. MEP amplitudes significantly decreased following 20 Hz tACS at time T1 and T2, in contrast with the sham stimulation. MEPs were significantly reduced in the 20 Hz tACS condition at time T2 in comparison with the 10 Hz tACS condition but did not significantly change at time point T1 between the 20 Hz and 10 Hz tACS conditions. There were no significant differences at time point T1 and T2 between the 10 Hz tACS condition and the sham stimulation. * denotes significant differences between groups (* *p* < 0.05).

**Figure 3:**
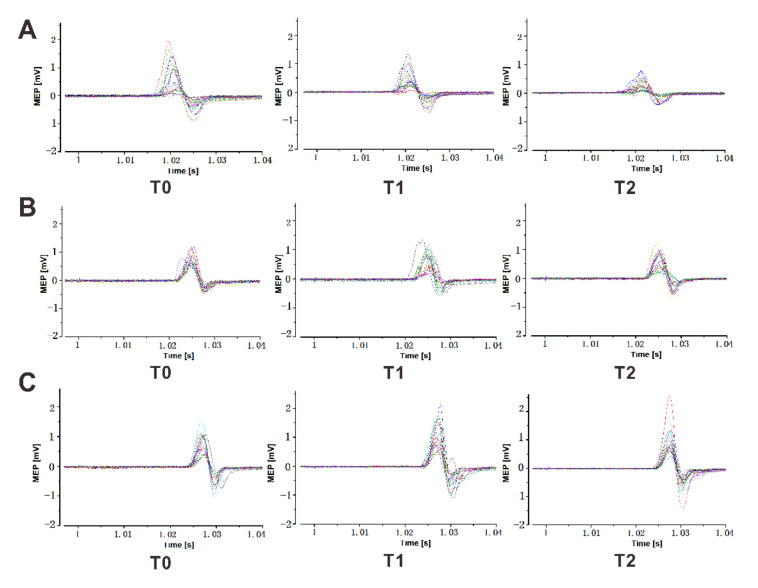
Raw electromyographic data traces from a representative individual showing motor-evoked potentials (MEPs). MEPs evoked by TMS over the left motor cortex in the 20 Hz tACS (**A**), 10 Hz tACS (**B**) or sham stimulation (**C**) conditions. Either tACS at 20 Hz and 10 Hz or a sham stimulation was applied over the left M1 during the action observation, followed by the action execution. MEPs were recorded before the action observation (T0), immediately after the action observation (T1), and after the action execution (T2).

The multi-level modeling approach showed a significant main effect of group (*F*_247.056_ = 5.387, *p* = 0.008), but not of time (*F*_258.193_ = 1.364, *p* = 0.264), and no significant interaction between time and group (*F*_455.737_ = 1.774, *p* = 0.147). The post-hoc tests showed that the MEP amplitudes were significantly smaller at time point T1 (after the action observation) for the 20 Hz tACS condition (*p* = 0.042, Cohen’s *d =* 0.611) in comparison with the sham stimulation, but did not significantly change between the 20 Hz and 10 Hz tACS conditions (*p* = 0.136, Cohen’s *d =* 0.536). The results further revealed a significantly smaller MEP amplitude at time point T2 (after the action execution) in the 20 Hz tACS condition, as compared to the sham stimulation (*p* = 0.0003, Cohen’s *d =* 0.852), and the 10 Hz tACS condition (*p* = 0.01, Cohen’s *d =* 0.622). No significant differences at time points T1 and T2 were observed between the 10 Hz tACS and the sham stimulation conditions (Figure 2). Representative examples of the EMG data traces and MEPs for a participant are shown in Figure 3.

## 4. Discussion

The main results of this study were that MEP amplitudes significantly decreased following 20 Hz tACS over M1 during the action observation and after the subsequent action execution. In contrast, no significant change of MEP amplitudes was observed in the 10 Hz tACS condition in comparison with the sham stimulation. 

Motor cortex excitability was not altered by the action observation, and execution, under sham stimulation. This outcome is in accordance with the results of other studies, which have shown that action observation alone does not modulate motor cortex excitability immediately [32,33,34]. Similarly, action observation followed by action execution did not enhance MEP amplitudes under sham stimulation conditions in a previous study by our group, in which the same task was applied [1]. The missing effect of alpha tACS on motor cortex excitability is in accordance with previous studies, in which alpha tACS (10 Hz) applied over the left M1 did not modulate cortical excitability [26,35,36]. One reason for these missing effects might be that MEPs measured by TMS over M1 mainly reflect changes of cortical excitability (facilitation and inhibition) related to motor pathways [37], whereas alpha oscillations originate mainly in postcentral regions and are more closely related to sensory processes [27]. In the present study, the participants were asked to perform action observation and execution that may have been more closely related to voluntary movement. Some studies, however, indicated that tACS at alpha frequency improved motor skill learning and consolidation [26,38]. It has been reported that alpha tACS over left M1 improved the consolidation of general motor skills and sequence-specific skills in elderly people, whereas it impaired sequence-specific skill consolidation in a serial reaction time task in young adults [38]. The effect of alpha tACS on motor process might therefore be age- and task-dependent. 

In comparison with those who received the sham stimulation, the participants who received beta tACS (20 Hz) showed diminished MEP amplitudes after action observation, and subsequent action execution. This result is in line with a previous study by Cappon et al. (2016) indicating that 10 min of 20 Hz tACS applied during a motor task resulted in decreased MEP amplitudes [19]. In further accordance, Feurra et al. (2013) showed that beta tACS decreased MEP amplitudes during motor imagery [39]. It is suggested that the efficacy of tACS on cortical activity depends on the state of the stimulated cortical area [40]. A 20 Hz tACS might have led to reduced desynchronization of local cortical oscillatory activity and thus diminished task-related activity. Similarly, Zaghi et al. (2010) reported an inhibitory effect of 15 Hz tACS applied for a duration of 20 min on motor cortex excitability [20]. The underlying mechanism might be that 20 Hz tACS at an intensity of 1 mA during action observation and subsequent action execution induces GABAergic activity augmentation, which contributes to the suppression of motor cortex excitability [12]. Another study by Wischnewski et al. (2019) indicated that N-methyl-D-aspartate receptor (NMDAR) mediated synaptic plasticity after applying 20 Hz tACS at an intensity of 2 mA in a resting state [23]. State-dependent influences and different intensity stimulations in tACS interventions may induce different levels of motor cortex excitability. The assumed entrainment of brain oscillations and reduction of cortical excitability following the intervention with beta tACS over the motor cortex may also modulate motor behavior and performance, although this was not explored in the present study, which might be used as a therapeutic tool for neurological disorders in clinical practice. The delivery of tACS at beta frequency over the primary motor cortex after motor skill acquisition facilitates consolidation and improves motor memory retention [41]. Beta tACS decreases the amplitude of repetitive finger-tapping and slows down voluntary movements in healthy subjects [42,43]. A presumed inhibitory effect of beta tACS on unwanted movements would suggest that it may suppress excessive or unintended outputs of the motor system, for instance, in tics or dyskinesias [10]. Accordingly, a case study demonstrated that 15 Hz tACS over the sensorimotor cortex reduced dystonic symptoms in a patient with idiopathic cervical dystonia [44]. 

A combined tACS-TMS approach was used to investigate the online effect of tACS over the M1. In the research of Pozdniakov et al. (2021), increased motor cortex excitability was detected during ongoing 20 Hz tACS stimulation in a resting state with no offline effects in both 10 Hz and 20 Hz tACS conditions [45]. It could be inferred that the effect of tACS was modulated by the time course of the stimulation’s administration (online vs. offline). Feurra et al. (2019) indicated a greater excitatory online effect of beta-tACS at rest than during action observation [46]. In the study of Cappon et al. (2016), an inhibitory offline effect on motor cortex excitability was observed when delivering 20 Hz tACS during a masked prime task [19]. This state-dependent effect has been found in other forms of non-invasive brain stimulation techniques. It has been reported that voluntary muscle contraction after transcranial direct current stimulation (tDCS) reduced or even tended to reverse the respective modulation effect of anodal or cathodal tDCS on motor cortex excitability [47]. In one of our previous studies, transcranial random noise stimulation (tRNS) applied during mirror-matched action observation increased motor cortex excitability, while no effect was found when tRNS was combined with action observation of mirror-reversed video, perceptual sequence video, or a landscape picture [1]. Combined brain stimulation and different motor tasks may induce variable neuroplastic alterations that should be used with caution in practical applications.

## 5. Limitations

This study has several limitations that should be mentioned. Motor performance was not tested in the present study, thus we cannot draw conclusions about tACS-induced behavioral changes, or about respective connections with the observed physiological effects. Moreover, we did not obtain data about tACS effects on brain oscillatory activity, and thus the underlying neurophysiological processes remain incompletely explored. Another limitation was that we did not include a group on which we performed a tACS intervention alone, so we were not able to compare the effect of tACS alone with the interaction between tACS and action observation on motor cortex excitability. A further limitation was that we studied the effects of tACS in healthy adults. If, and to what extent, the current findings apply to patients with neurological or motor disorders remains to be investigated in future studies.

## 6. Conclusions

Transcranial alternating current stimulation at 20 Hz but not 10 Hz during action observation inhibited motor cortex excitability, and this effect remained after subsequent action execution. The influence of tACS on task-related motor cortex excitability is thus frequency-dependent. The current findings can help to develop therapeutic applications in patients with neurological or psychiatric diseases involving pathologically enhanced motor behavior-related cortical excitability.

## Data Availability

The data presented in this study are available on request from the corresponding author.
